# Navigating the Balance: Treatment-Resistant Schizophrenia Relapse Risks Versus Clozapine-Related Cardiovascular Complications - a Case Report

**DOI:** 10.1192/bjo.2024.689

**Published:** 2024-08-01

**Authors:** Hardeep Singh, Alexandra Blackman

**Affiliations:** Surrey and Borders NHS Foundation Trust, Surrey, United Kingdom

## Abstract

**Aims:**

Clozapine, known for its efficacy in treating treatment-resistant schizophrenia, offers significant benefits. However, its use also carries potential cardiovascular side effects, such as myocarditis early in treatment and cardiomyopathy with prolonged use. This case highlights the challenge of balancing the risks of treatment-resistant schizophrenia relapse against the potential cardiovascular complications associated with clozapine therapy.

**Methods:**

An adult male with treatment-resistant schizophrenia was initially prescribed clozapine but switched to paliperidone depot due to compliance issues. However, he relapsed shortly after and had to be restarted on clozapine, albeit at a lower dose due to associated tachycardia, supplemented with risperidone. After two years on clozapine, he was diagnosed with cardiomyopathy, prompting a cardiology review. Clozapine was withheld, and risperidone dosage was increased, but he experienced a severe relapse. Despite the risks, multiple multidisciplinary meetings and approval from the medication optimization committee led to his re-commencement on clozapine due to the treatment-resistant nature of his illness and associated risks.

**Results:**

Clozapine, a benzisoxazole derivative, is used for treatment-resistant schizophrenia and aggressive behaviours. Its pharmacological action involves D2 and 5HT2A receptor antagonism, affecting serotonergic, dopaminergic, adrenergic, cholinergic, and histaminergic receptors. However, severe side effects like agranulocytosis, seizures, myocarditis, tachycardia, and cardiomyopathy can occur. Cardiomyopathy incidence is rare (0.02–0.1%) with a mortality rate of 17.9%. Proposed mechanisms include undetected myocarditis and persistent tachycardia-induced changes leading to ventricular dysfunction. Common findings in investigations include raised CRP, leucocytosis, eosinophilia, increased lactate, elevated troponin, non-specific ECG changes, and ventricular dysfunction on echocardiography.

**Conclusion:**

Clozapine poses rare but potentially fatal cardiac risks, including myocarditis and cardiomyopathy. Essential baseline investigations and close monitoring during the initial weeks are crucial. Persistent tachycardia may signal trouble. If suspected, serial monitoring of FBC, troponin, and CRP levels is recommended, with prompt management if confirmed with discontinuation of clozapine, as the cardiomyopathy is often reversible. A multidisciplinary approach involving cardiology is vital for optimal management. This is particularly crucial when weighing the risks of relapse in schizophrenia against the potential cardiovascular complications of clozapine therapy.

